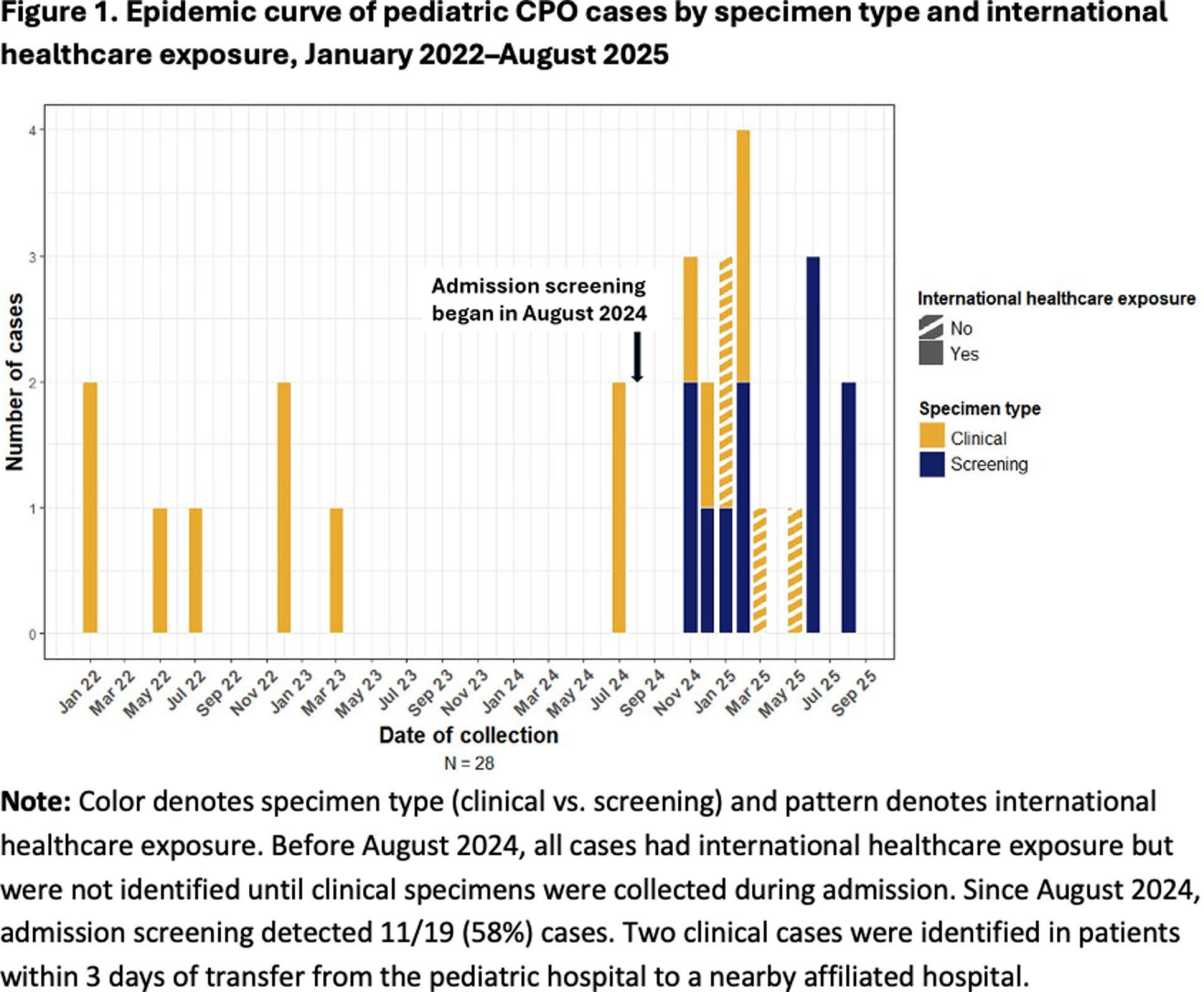# 346 Shortening the Duration of Contact Isolation for Multidrug-Resistant Organisms in a Pediatric Hospital: A Quality Improvement Project

**DOI:** 10.1017/ash.2026.10686

**Published:** 2026-06-23

**Authors:** Lian Hsiao, Sandeep Sangha, Pamela Abdali, Sydney Loewen, Kiara Velasquez, Liz Mason, Daniel Dodson, Sam Horwich-Scholefield, Erin Epson, Tisha Mitsunaga

**Affiliations:** 1 California Department of Public Health, Healthcare-Associated Infections Program; 2 Shriners’ Childrens Northen California; 3 Sacramento County DHS; 4 California Department of Public Health; 5 CDPH

## Abstract

**Background:** Carbapenemase-producing organisms (CPOs) can cause serious, difficult-to-treat infections and spread rapidly in healthcare settings. Although pediatric CPO cases are uncommon, they may be increasing in the United States. We describe CPO surveillance practices and patient and specimen characteristics among patients admitted to a California pediatric specialty hospital that regularly receives high-acuity patients referred from healthcare facilities outside the US. **Methods:** Carbapenem-resistant organisms identified from clinical specimens underwent molecular testing to identify the “big-5” carbapenemases (i.e., KPC, NDM, OXA-48 like, VIM, IMP); carbapenem-resistant Acinetobacter baumannii (CRAB) isolates were also tested for additional oxacillinase (OXA) variant carbapenemases at a public health laboratory. In August 2024, the hospital began screening patients upon admission from non-US healthcare facilities by collecting rectal swabs for molecular testing for big-5 carbapenemases, and culture-based screening for CRAB with molecular testing to identify the big-5 and additional OXA variant carbapenemases. We collected patient and specimen data from the hospital and public health case reports. We defined a case as carbapenemase(s) detected in a clinical or screening specimen from a pediatric patient (ages 1–18 years), either during admission or within 3 days of transfer from the hospital from January 2022 to August 2025. **Result:** Among 28 total cases, 17 (61%) were in clinical specimens (10 wound, 3 blood, 3 respiratory, and 1 tissue) and 11 (39%) in 34 rectal swab specimens (32% positivity) collected through admission screening starting in August 2024 (Figure 1). The most common carbapenemases were OXA-23-like (32%) and OXA-24/40-like (32%) and the most common organism was A. baumannii (79%). Of the 27 patients (one had two cases), the median age was 11 years; 20 (74%) were Hispanic and 21 (78%) were non-US residents. Twenty-two (82%) were admitted for burn injuries and 24 (89%) had international healthcare exposure. **Conclusion:** Admission screening based on international healthcare exposures had a high (32%) yield and identified more than half of all CPO cases since implementation. The majority of CPO cases were OXA variant-producing CRAB, highlighting the role of culture-based screening to identify carbapenemase-producing CRAB that otherwise would not be detected by screening with molecular testing for big-5 carbapenemases only. Risk-based admission screening is a key component of a comprehensive CPO prevention strategy by facilitating rapid detection of cases and timely implementation of focused infection prevention and control measures to prevent further spread among a vulnerable population.

**Figure:**